# Brain Serotonin Synthesis in Adult Males Characterized by Physical Aggression during Childhood: A 21-Year Longitudinal Study

**DOI:** 10.1371/journal.pone.0011255

**Published:** 2010-06-22

**Authors:** Linda Booij, Richard E. Tremblay, Marco Leyton, Jean R. Séguin, Frank Vitaro, Paul Gravel, Elisabeth Perreau-Linck, Mélissa L. Lévesque, France Durand, Mirko Diksic, Gustavo Turecki, Chawki Benkelfat

**Affiliations:** 1 Department of Psychiatry, McGill University, Montreal, Canada; 2 Departments of Psychology and Pediatrics, University of Montreal, Montreal, Canada; 3 School of Public Health and Population Sciences, University College, Dublin, Ireland; 4 INSERM U669, Paris, France; 5 Sainte-Justine Hospital Research Center, Montreal, Canada; 6 Department of Psychiatry, University of Montreal, Montreal, Canada; 7 Department of Neurology and Neurosurgery, Montreal Neurological Institute, McGill University, Montreal, Canada; 8 McConnell Brain Imaging Center, Montreal Neurological Institute, McGill University, Montreal, Canada; 9 School of Psycho-Education, University of Montreal, Montreal, Canada; 10 McGill Group for Suicide Studies, Douglas Hospital, McGill University, Montreal, Canada; University of Wuerzburg, Germany

## Abstract

**Background:**

Adults exhibiting severe impulsive and aggressive behaviors have multiple indices of low serotonin (5-HT) neurotransmission. It remains unclear though whether low 5-HT mediates the behavior or instead reflects a pre-existing vulnerability trait.

**Methodology/Principal Findings:**

In the present study, positron emission tomography with the tracer alpha-[^11^C]methyl-L-tryptophan (^11^C-AMT) was used to compare 5-HT synthesis capacity in two groups of adult males from a 21-year longitudinal study (mean age ± SD: 27.1±0.7): individuals with a history of childhood-limited high physical aggression (C-LHPA; *N* = 8) and individuals with normal (low) patterns of physical aggression (LPA; *N* = 18). The C-LHPA males had significantly lower trapping of ^11^C-AMT bilaterally in the orbitofrontal cortex and self-reported more impulsiveness. Despite this, in adulthood there were no group differences in plasma tryptophan levels, genotyping, aggression, emotional intelligence, working memory, computerized measures of impulsivity, psychosocial functioning/adjustment, and personal and family history of mood and substance abuse disorders.

**Conclusions/Significance:**

These results force a re-examination of the low 5-HT hypothesis as central in the biology of violence. They suggest that low 5-HT does not mediate current behavior and should be considered a vulnerability factor for impulsive-aggressive behavior that may or may not be expressed depending on other biological factors, experience, and environmental support during development.

## Introduction

In contrast to popular belief, longitudinal epidemiological research indicates that the sudden onset of physical aggression in adolescence is unusual [1]. Rather, physically aggressive behaviors can already be detected by 12 months of age, and their frequency peaks between the end of the second and fourth years [1], [2]. In the majority of children, the frequency of physical aggression gradually decreases, starting before elementary school entry [1], [3]. However, others start desisting only at the end of elementary school and others not until the end of adolescence [4]. A 60-year longitudinal study of juvenile delinquents concluded that very few show life-span high frequency of violent offending [5]. Among those who do express chronic physical aggression, impaired executive functioning is evident in adolescence and early adulthood, even after controlling for other cognitive-neuropsychological domains, intelligence quotient (IQ) and Attention Deficit Hyperactivity Disorder (ADHD) symptoms [4], [6], [7], [8]. Longitudinal follow up of elementary school children with high levels of physical aggression demonstrates that they are at greater risk for substance abuse, anti-social personality, suicide, depression, spouse abuse and neglectful and abusive parenting [9], [10], [11]. However, the long-term outcomes for the physically aggressive children who desist with adolescence have not been studied [12], [13].

Genetic epidemiology studies of aggressive behavior demonstrate that childhood physical aggression is highly heritable [14], [15], [16]. The neurobiological substrates underlying the individual variability in the development of physical aggression and violence are many, although there seems to be little agreement about their relative importance [17]. Generally, heuristic models support the view that gene by environment interactions manifest their effects through changes in brain structure and/or function, resulting into deficits in cortical *top down* control and/or facilitation of *bottom up* signalling triggered from limbic circuits [18]. One proposed mechanism contributing to reduced *top down* control and behavioral disinhibition is lower brain serotonin (5-HT) neurotransmission in the orbitofrontal cortex (OBFC) [18].

The present study is part of a series of studies that applied a significant paradigm shift, from clinical studies of patient samples to a developmental epidemiological and longitudinal approach. Bio-psycho-social factors and outcomes were investigated with a population-based longitudinal cohort of young males from low socioeconomic area schools in Montreal (Canada) [19]. More specifically, the current study tested the hypothesis that adult males who were on a high physical aggression trajectory during elementary school and desisted during adolescence (childhood-limited high physical aggression, C-LHPA) still maintained lower regional brain 5-HT synthesis during adulthood compared to males who had a more normal pattern (low) of physical aggression (LPA) [3]. Based on results from previous neuro-imaging studies in animals and in patients with impulsive-aggressive behaviors [20], [21], the OBFC was chosen as primary volume of interest (VOI). All participants were assessed clinically, with a particular emphasis on current and recent impulsive aggressive behavior, frontal lobe function, emotional intelligence and psychosocial adjustment. Genotyping for the rate-limiting 5-HT synthesis enzyme, tryptophan-hydroxylase2 (*TPH_2_*) and prospective measures of early and late life adversity were also collected in order to explore genetic and environmental influences on 5-HT metabolism. Brain 5-HT synthesis was estimated by means of positron emission tomography (PET) in combination with the injection of the synthetic analog of the 5-HT precursor tryptophan, alpha-[^11^C]methyl-L-tryptophan (^11^C-AMT) [22], [23], [24].

## Results

### PET/^11^C-AMT

**Figure 1:**
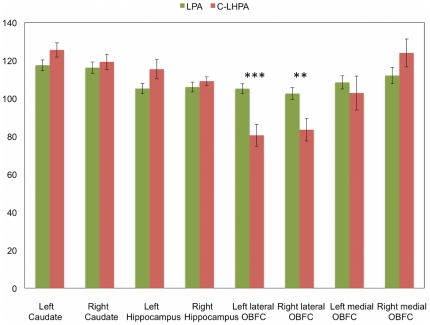
Normalized K* values, as a function of VOI and group. Values represents the mean ± s.e.m. of the high *vs*. low physical aggression developmental trajectory) (*N* = 26). *** *P*<0.001; ** *P*<0.01.

Results of the VOI analysis are displayed in **Figure 1**. There was a multivariate effect of aggression trajectory (*F*(8,17) = 5.28, *P* = 0.002; partial eta squared 0.71). Univariate statistics (General Linear Models (GLM) for MANOVA) showed that the C-LHPA group had lower normalized K* values in the left (*F*(1,24) = 22.61, *P*<0.001; partial eta squared:0.485) and right lateral OBFC (*F*(1,24) = 11.20, *P* = 0.003; partial eta squared: 0.32), relative to the LPA group. There were no other significant differences in normalized K* values for any of the other selected VOIs.

**Figure 2:**
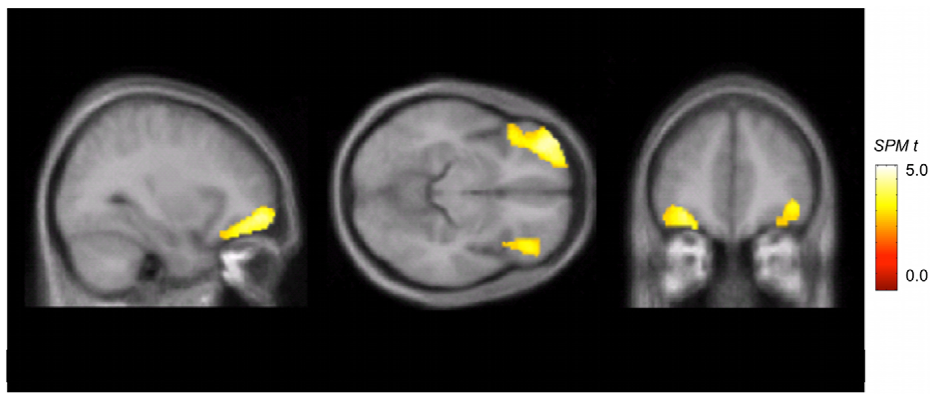
Statistical Parametric Mapping of ^11^C-AMT Plasma-to-Brain clearance. The figure represents a comparison between young adults with a high physical aggression trajectory during elementary school and desisted during adolescence (C-LHPA) *vs*. those who had a more normal pattern (low) of physical aggression (LPA) (*N* = 26). The color-coded images indicate a marked decrease in brain serotonin synthesis capacity bilaterally in the OBFC in those subjects with the high physical aggression trajectory.

Statistical Parametric Mapping (SPM) results are displayed in **Figure 2** **.** Consistent with the VOI analysis, the C-LHPA group demonstrated lower normalized K* values in the left and right OBFC and the inferior and middle frontal gyrus (max voxels = 1472, Peak T = 5.87, Z = 4.58; *P*<0.001, uncorrected, *P* = 0.06, corrected) (Brodmann Area (BA) 11, BA 47). In addition, the SPM analysis identified in the C-LHPA group areas of increased normalized K* in limbic areas, including the insula (max voxels = 1215, Peak T = 5.93, Z = 4.61; *P*<0.001 uncorrected, *P* = 0.06, corrected), and the parahippocampal gyrus (max voxels = 152, Peak T = 5.83, Z = 4.56; *P*<0.001, uncorrected, *P* = 0.06, corrected).

### Free plasma Tryptophan

There were no group differences in free tryptophan levels (mean ± s.e.m. LPA *vs*. C-LHPA group: 10.4±0.7 *vs*. 9.5±0.8) (*F*(1,24) = 0.64, *P* = 0.43).

### *TPH_2_*

There were no group differences in allelic distribution of the *TPH_2_* polymorphic variants examined: rs4641527 (*P* = 0.38); rs2129575 (*P* = 0.64); rs1023990 (*P* = 0.31); rs6582071 (*P* = 0.27) (all *F*-exact, 1 tailed).

### Aggression and impulsivity measures in adulthood

A significant difference in the expected direction was observed between the C-LHPA and LPA groups on a retrospective measure of aggression in childhood and adolescence, but no significant difference was observed for current aggression (Table 1). However, the C-LHPA group exhibited significantly higher self-reported scores for impulsivity (Barratt Impulsivity Scale; BIS). Nonetheless, the two groups did not differ for the total number of commission errors (CE), nor for the number of CE in the Reward-Punishment component of the Go/NoGo task, a measure previously reported to be increased in clinical samples endowed with high impulsivity and/or aggression [25], [26], [27].

**Table 1:** Characteristics of the sample.

Variable	LPA (*n* = 18)	C-LHPA (*n* = 8)	Group
*Total score Beck Depression Inventory* (s.e.m.)	2.94(0.9)	3.38(1.2)	*F*(1,24) = 0.09, *P* = 0.76
*Personal and family history of Psychopathology:*			
- History of substance abuse (%)	22.2%	37.5%	*F* Exact, 2 tailed: 0.63
- History of mood disorders (%)	0%	25.0%	*F* Exact, 2 tailed: 0.09
- First-degree relative with mood disorders (%)	22.2%	0.0%	*F* exact, 2 tailed: 0.28
- First-degree relative with substance abuse (%)	33.3%	25.0%	*F* Exact, 2 tailed: 1.00
*Brown-Goodwin History of Violence:*			
- Childhood/Adolescence (s.e.m.)	7.4(1.6)	19.4(3.9)	*F*(1,24) = 13.31, *P* = 0.001
- Adulthood (s.e.m.)	3.9 (0.6)	4.9(1.0)	*F*(1,24) = 0.58, *P* = 0.45
*Barratt Impulsiveness Scale* (s.e.m.)	55.6(2.2)	65.6(4.1)	*F*(1,24) = 4.81, *P* = 0.04
*Go/NoGo task:*			
- Total number of CE (s.e.m.)	41.7 (7.1)	40.2(13)	*F*(1,23) = 0, *P*>0.99
- # of CE reward-punishment condition (s.e.m.)	11.2(2.4)	7.1(2.6)	*F*(1,23) = 0.64, *P* = 0.43
* Self-Ordered Pointing Task (SOP)*			
- Concrete trials (s.e.m.)	1.78(0.5)	1.5(0.8)	*F*(1,24) = 0.06, *P* = 0.81
- Abstract trials (s.e.m.)	2.17(0.4)	2.0(0.6)	*F*(1,24) = 0.06, *P* = 0.80
* Spatial Conditioned-Association Task (SCAT)*			
- Completed (s.e.m.)	91.8 (11.3)	101.1(17)	*F*(1,24) = 0.24, *P* = 0.63
- # of errors (s.e.m.)	62.9(14.6)	97.1(37)	*F*(1,24) = 1.05, *P* = 0.32
- # of incomplete trials (s.e.m.)	30.9(5.9)	39(11.4)	*F*(1,24) = 0.44, *P* = 0.51
*Mayer–Salovey–Caruso Emotional Intelligence Test (MSCEIT)*			
-Experiential	101.6(2.3)	101.9(2.7)	*F*(1,24) = 0, *P* = 0.99
-Strategic	91.7(1.9)	94.2(2.4)	*F*(1,24) = 0.40, *P* = 0.53
Total	95.8(2.2)	98.2(2.8)	*F*(1,24) = 0.28, *P* = 0.60
* Bar-On Emotional Intelligence Total quotient*	105.1(3)	102.4(5.3)	*F*(1,24) = 0.17, *P* = 0.68

**Note.** Table represents the raw (uncorrected) means (s.e.m.) (*N* = 26). *F*-values are corrected for probability of trajectory (see methods section). The Go/NoGo task was missing for one participant in the LPA group.

LPA =  Normal development of low physical aggression.

C-LHPA =  Childhood-limited high physical aggression.

Given the finding in the C-LHPA group of higher self-reported impulsiveness relative to the LPA group, and studies showing that low 5-HT neurotransmission is a biological risk factor for both aggressive behaviors and behavioral disinhibition, (e.g. [18], [28]), the primary VOI analysis was rerun, including the BIS score as a covariate. The results of aggression trajectory on normalized K* in the OBFC were only slightly attenuated, indicating a multivariate effect of aggression trajectory (*F*(8,16) = 3.82, *P* = 0.01; partial eta squared: 0.66), and univariate statistics showing lower normalized K* in the left lateral OBFC (*F*(1,23) = 15.68, *P* = 0.001; partial eta squared: 0.41) and in the right lateral OBFC (*F*(1,23) = 6.29, *P* = 0.02; partial eta squared: 0.22) in the C-LHPA group, relative to the LPA group.

### Personal and family history of psychopathology/Working memory/Emotional Intelligence

None of the participants met the criteria for any axis I or II disorder. Neither of the two groups differed on family or personal history of mood and substance abuse, nor did their performance differ on working memory (Self-Ordered Pointing task (SOP) [29] and the Spatial Conditioned-Association task (SCAT) [30]) and emotional intelligence (BarOn Emotional Quotient Inventory (EQ-I) [31], [32] and Mayer-Salovey-Caruso Emotional Intelligence Test (MSCEIT) [31], [32] (Table 1).

### Family adversity during childhood and adult psychosocial status

The groups did not differ in overall family adversity during childhood and on measures of current psychosocial variables, including civil status, number of children, health, education level, job status, average salary, number and quality of relationships, criminal status, and quality of intimate relationships (Table S1).

## Discussion

The present study investigated brain 5-HT synthesis in a community sample of healthy adult males characterized by high levels of physical aggression during the primary school years and a desisting pattern during adolescence (C-LHPA). We compared them to males recruited from the same cohort with a normal (low) physical aggression trajectory during childhood and adolescence (LPA). We found no differences in aggressive behavior, mood state, social status and neuropsychological measures of working memory, emotional intelligence and impulsivity between the C-LHPA and LPA groups, yet the C-LHPA group demonstrated markedly lower 5-HT synthesis in the bilateral OBFC in adulthood relative to those with a normal aggression trajectory. In fact, the magnitude of the reduction of ^11^C-AMT uptake and trapping in the OBFC was about twice the one previously reported in patient samples with impulse control disorders [27], [33]. This observation forces a re-examination of the low 5-HT hypothesis as a central tenet of the biology of violence.

5-HT plays a significant role in neurodevelopment. In humans, 5-HT neurons are constituted approximately 5 weeks after gestation [34], 5-HT fibers grow in the cortex prenatally, and within a short period of time, between 12–14 weeks of gestation, the thalamocortical axons transiently express the 5-HT transporter, crucial for the fine-tuning of cortical development [35]. 5-HT levels continue to increase throughout the first two to five years of life and then gradually decrease until adult levels are reached (approximately age 14) to a level approximating 50% of the peak values of early childhood [36]. Research in mice has shown that altered 5-HT availability during a critical window of early development, through genetic or pharmacological manipulations and/or environmental events, can result in structural and/or functional alterations in brain neurotransmission with deleterious consequences on emotion regulation in adulthood [37], [38]. We presume that relatively low 5-HT synthesis capacity partially accounts for high levels of physical aggression in childhood. However, the substantial decrease in frequency of physical aggression during adolescence, as observed in our C-LHPA group, cannot be directly attributed to a relative increase in 5-HT synthesis capacity, at least not from the level observed at 27 years of age. We suggest that low brain 5-HT in the C-LHPA group may operate as a vulnerability trait, but other protective environmental factors, as well as brain maturation and new learning, may have come into play with adolescence and early adulthood to support impulse control despite low 5-HT. Indeed, a 20 year longitudinal study of pre-pubertal adolescents examining sensation-seeking and impulsivity found that by age 15, both measures demonstrated a steady linear decline, fitting well with the developmental pattern of other self-regulatory tasks that tap self-regulation (Go/NoGo, Stroop, Antisaccades), as well as the maturational time course of the brain circuits that subserve impulse control [39]. Consistent with this study, many of the differences in the behavioral phenotype noted between our two groups in childhood had disappeared at the 21 year follow-up; and both groups were indistinguishable on the basis of psychosocial status in adulthood. We suggest that, while 5-HT neurotransmission tends to remain stable over time, from childhood to adulthood [40], experience, brain maturation and/or environmental support play a major role in the near universal reduction in frequency of physical aggression with age, even in the most delinquent males [5].

The validity and significance of the observations presented in this study, however, rest upon the following considerations: **(1)** When the technique was originally implemented, it was argued that the brain regional uptake of ^11^C-AMT might reflect blood-brain transport rather than brain 5-HT trapping and thereby, 5-HT synthesis [41]. Experimental evidence accumulated over more than 10 years [23], [42], [43], [44], [45], [46] now provides a firm basis for a consensus that brain regional ^11^C-AMT trapping represents an acceptable index of 5-HT synthesis. Of particular interest is the report of Patlak plots of ^11^C-AMT brain uptake estimated in primates, with a slope significantly different from zero, and providing unequivocal evidence for brain trapping of the tracer [24]. **(2)** The study was conducted between 2002 and 2007. An older version of the SPM software (SPM99) was used for exploratory image analysis. However, reanalysis of the image data with a newer version of SPM (SPM2) yielded similar results. Newer versions of SPM have now been released (SPM5 and SPM8), although much of the add-on functionality(ies) are for functional Magnetic Resonance Imaging (fMRI) analysis. The use of pre-processing steps (co-registration/realignment) relying on tools developed and validated in-house (MINC tools) at the Montreal Neurological Institute (MNI) has made the use of newer versions of SPM less critical for PET investigations. **(3)** The interpretation of the results rests upon the assumption that normalized K* is a stable measure over time [47]. Cerebrospinal fluid (CSF) concentrations of 5-Hydroxyindoleacetic acid (5-HIAA) in humans and monkeys are highly correlated over time [40]. In addition, in humans, low levels of CSF 5-HIAA have already been found in infants (age between birth and 3 months) from parents with anti-social personally disorders [48]. These studies support the idea that lower 5-HT synthesis in young adults as observed in the present study would most likely indicate a neurochemical vulnerability present since childhood. **(4)** Our study was limited to males. The developmental pattern of aggressive behavior [49], as well as many aspects of brain 5-HTneurotransmission, including ^11^C-AMT uptake and trapping, are influenced by gender [22], it would be of much interest to investigate the extent to which the present results generalize to females. **(5)** 5-HT neurotransmission exercises an inhibitory tonic control on behavior; low central 5-HT facilitates behavioral disinhibition and its most extreme clinical manifestation, impulsive aggression [50], [51]. Whether aggressive behavior at school reflected increased impulsivity was not specifically tested at that age. This was however, unlikely, since measures of impulsivity collected in the laboratory in adolescence and during early adulthood, by and large, did not discriminate between trajectories. Furthermore, co-varying out the PET data for BIS scores measured in adulthood in both trajectories, did not affect the results.

In summary, this study compared brain regional ^11^C-AMT uptake and trapping, a proxy measure of 5-HT synthesis, in a group of young adult males on a childhood-limited high physical aggression trajectory and in a group of young males from the same population who were on a normal (low) trajectory of physical aggression during childhood and adolescence. As predicted, the C-LHPA group exhibited lower ^11^C-AMT in the OBFC, with an effect size about twice of what was previously reported in patient samples endowed with an impulsive aggression phenotype. Surprisingly however, in the presence of relatively low 5-HT synthesis in the C-LHPA group, neither groups differed markedly in adulthood in their levels of aggressive behavior, nor in their behavioral, neurocognitive and psychosocial outcomes. This observation is interpreted as reflecting the mitigating effects of brain maturation, learning and supportive environment in the case of participants from the high aggression “desisters” trajectory. Altogether, these data emphasize a less deterministic model with a role for familial and other environmental factors operating at different developmental time periods. More generally, these observations may lead to a re-examination of the low 5-HT diathesis theory, as a risk factor in the pathogenesis of impulsive aggression.

## Materials and Methods

### Ethics Statement

All participants provided written informed consent. The study was carried out in accordance with the Declaration of Helsinki, and was approved by the research ethics committee at the MNI.

### Participants

Adult males (mean age ± SD = 27.1±0.7) were recruited from a longitudinal study of a French-speaking community sample (*N* = 1,037) attending one of the 53 schools in disadvantaged areas of Montreal. Teachers assessed these boys' physical aggressive behavior first at age 6, then every year from age 10 to 15 with the Teacher Form of a French-Canadian version of the Social Behavior Questionnaire [3].

**Figure 3:**
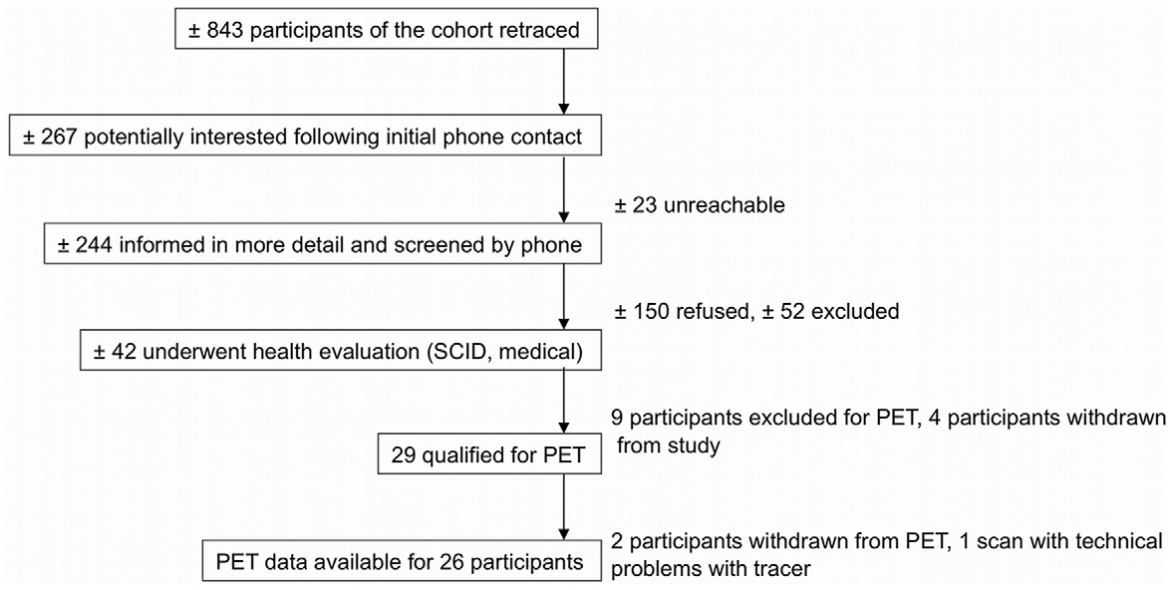
Schematic overview of the recruitment process.

A schematic overview of the recruitment is illustrated in **Figure 3**. Following a psychiatric assessment (Structured Clinical Interview for DSM-IV: Patient Edition (SCID-P; [52]; and the Structured Clinical Interview for DSM-IV Axis II Personality Disorders (SCID-II) [53] if there were any indications during the psychiatric assessment of the presence of an axis II disorder), the Beck Depression Inventory [54], determination of the presence/absence of psychiatric/medical illness in the family by construction of family pedigrees and a complete physical examination and routine laboratory testing (glucose, electrolytes, alanine transaminase, aspartate aminotransferase, creatinine, thyroxine, thyroid-stimulating hormone, complete blood count, Electrocardiography), neuropsychological testing was conducted in 32 individuals. PET data were obtained in 29 of them. Three out of the 32 participants disqualified for PET because of the report of past head trauma (*n* = 2) or having an extensive history of brain tumors in the family (*n* = 1). Of the 29 participants that qualified for PET, two of them were not scanned because of technical problems with the tracer production and they could not be rescheduled. Another participant was scanned, but the acquired data were invalid because of technical problems with the tracer. Hence, complete data were available for 26 individuals. For consistency purposes, only the results of the 26 participants for whom complete data were available are presented.

Main exclusion criteria were: major medical/neurological illness likely to confound PET analysis or results, including cushing disease, epilepsy, stroke, dementia, history of head trauma with loss of consciousness; testing positive on a urine toxicology screen for illicit drugs of abuse on the day of the study (Phencyclidine (PCP), Tetrahydrocannabinol (THC), Amphetamine, Cocaine, Benzodiazepines, opiates); history of exposure to 3,4-Methylenedioxy-methamphetamine (MDMA) in the past month; current diagnosis of alcohol/drug abuse or dependence; current diagnosis of mood disorders; current medication likely to affect the function of the central nervous system (CNS); general Magnetic Resonance Imaging (MRI) and PET exclusion criteria.

### Assignment of trajectories

Based on the measurements collected from childhood until age 15, four trajectories for physical aggression were previously identified for the entire sample of the longitudinal study (*N* = 1,037;[3]). One trajectory showed no physical aggression at any time points (never); a second trajectory started with moderate-low rates, which declined over time (low level desister); a third trajectory began with high rates of the behavior, which subsequently declined between 10 and 15 years (childhood-limited high); a final trajectory representing 3% of the entire cohort consisted of individuals who had consistently high physical aggression levels until age 15 (chronic). For the sake of the present study the childhood-limited high group was compared with the two low level groups (never/low level ‘desister’). Previous studies with this cohort have shown that the never/low-desister group is distinct from the higher aggression groups in terms of cognitive function, behavior and psychosocial characteristics [8], [13]. Albeit from distinct behavioral developmental trajectories since childhood, all participants in the present study were healthy adult males living in the community.

### Positron Emission Tomography (PET) and Magnetic Resonance (MR) Imaging

All participants underwent a sixty-minute dynamic PET scan, conducted with an ECAT HR+ scanner (ECAT HR+; CTI Molecular Imaging, Inc/Siemens, Knoxville, Tennessee). Transmission scans for attenuation correction were performed using a ^68^Ge/Ga source prior to tracer injection. Immediately after the intravenous injection of 10 mCi (370 MBq) of alpha-[^11^C]AMT injected over a 2 minutes period, 60-minute dynamic PET data were acquired. The advantages and disadvantages of the method have been described previously [45]. Overall, a general consensus is emerging that brain regional ^11^C-AMT trapping provides an acceptable proxy for 5-HT synthesis [23], [24], [45], [55]. All images were collected and reconstructed using a 3-dimensional mode with an intrinsic resolution of 5×5×5 mm full width at half maximum, with 26 frames, each with 128×128 voxels and 63 slices and a voxel size of 2.4×2×2 mm. Blood samples were drawn throughout the PET scan from the antecubital vein at regular time intervals of progressively longer duration, in order to plot the ^11^C-AMT plasma time activity curves. The validation of using venous samples as an input function for the estimation of the rate of the 5-HT synthesis with PET ^11^C-AMT has previously been demonstrated [56]. All participants also underwent high-resolution magnetic resonance imaging using a 1.5-T superconducting magnet system (Philips Gyroscan; Philips Medical Systems, Eindhoven, the Netherlands) for the purpose of PET/MRI co-registration. Magnetic resonance imaging data were stored as a 256×256×160–mm matrix with 1-mm3 isotropic voxels.

### Determination of free tryptophan plasma levels

Three 2-ml venous blood samples were drawn from each individual during the PET study. The samples were centrifuged, and the ultrafiltrate was stored at −80°C for measurement of plasma free tryptophan using high-performance liquid chromatography.

### Genotyping

We recently reported that polymorphic variants in the *TPH_2_* gene (rs4641527; rs2129575; rs1023990; rs6582071), responsible for human brain synthesis, predicted subtle variations in 5-HT synthesis in the OBFC in a mixed sample of healthy volunteers and patients [57]. In order to control for the influence of these *TPH_2_* polymorphisms on brain 5-HT synthesis when comparing the high and low physical aggression groups, differences in genotype distribution for each of these *TPH_2_* variants between the trajectories were investigated. Genomic DNA was extracted from blood and analyzed according to standard procedures [58].

### Behavior and Cognition

The 11-item Brown-Goodwin History of Violence [59] was administered. Each item of the Brown Goodwin History of Violence was rated on a 4 points scale and was assessed for both childhood (<12 years), adolescence (12-18 years) and adulthood (>18 years). Measures of trait impulsivity in adulthood included the Barratt Impulsivity Scale (BIS) and the Go/NoGo task. The primary outcome variable of the Go/NoGo task was the total number of commission errors (CEs), reflecting continued responding despite negative feedback (loss of 10¢) and the number of commission errors in a reward punishment condition [25]. We have reported previously that Go/NoGo CEs are elevated in Borderline Personality Disorder (BPD), patients characterized by relatively low rates of brain 5-HT synthesis [27]. Measures of working memory included the Self-Ordered Pointing test (SOP) [29] and the Spatial Conditioned-Association task (SCAT) [30]. The BarOn Emotional Quotient Inventory (EQ-I); and the Mayer-Salovey-Caruso Emotional Intelligence Test (MSCEIT) [31], [32] were completed as measures of emotional intelligence.

### Adversity in Childhood and Adolescence

The index of *family adversity* is a composite score of the degree of adversity in families ranging from 0 to 1, used in previous studies with this cohort [60], [61], [62]: it consists of mother's and father's occupational prestige, mother's and father's age at birth of their first child, mother's and father's education level, and familial status. Family adversity was measured at age 6, and again at ages 10–16.

### Psychosocial Adjustment

Group differences in current psychosocial functioning were measured by interview prior to participation and by items extracted from the ‘Questionnaire about the development of the young adult’, a self-report follow up survey completed by the majority of the original cohort (*N* = 536) around the same time as the current study. Twenty-one of the participants who underwent the PET scan completed this survey. Groups were compared on their responses to questions about their current health, work and education, social life and relationships.

### VOI Analysis

Functional neuroanatomical mapping of brain structures and pathways involved in the expression of the diverse components of the impulsive aggression phenotype, regularly emphasizes the importance of cortical (OBFC), subcortical (caudate) and limbic (hippocampus, amygdala) areas, together with inter-related connections [18]. Lower 5-HT function in the OBFC is perhaps the most widely reported in the literature; hence, our selection of the OBFC as primary VOI in the current study. The hippocampus is among the most densely innervated limbic terminal fields of 5-HT fibers [34]. Hippocampal dysfunction (s) have been reported in aggressive behaviors [18], and in particularly in relation to early environmental adversity [63]. Finally, the caudate was selected as one of the primary VOI given its high density innervation in 5-HT neurons, estimated at up to 1–3 times greater than in the human frontal cortex [34], [64]. These regions have all been shown previously to be large enough to be reliably identified on each participant MRI, using an automatic segmentation method [65].

### Behavioral Statistics/VOIs

Chi-square statistics and (M)ANOVAs were used to investigate group differences on the outcome variables, including developmental aggression (high-level desister *vs*. low/absent) as between participants factor. For all of these GLM models, participants were statistically weighted according to the posterior probability of belonging to the assigned trajectory [3]. Representativeness of the sample for the entire cohort in terms of demographic/psychosocial background was investigated by comparable GLM models, including “current study participation” as a between participants factor.

### SPM analyses

Parametric K* images of ^11^C-AMT were generated [66], [67] and resampled into MNI305 2-mm isotropic stereotaxic space using a standard automatic algorithm [65]. The images were subsequently smoothed to a 14 mm resolution FWHM using an isotropic Gaussian filter. In order to cancel out global effects on regional K* values, regional K* values were normalized by the mean global K* of the gray matter to 100. For each of the investigated SNP, voxel-by-voxel group comparisons were made using SPM99 (Welcome Functional Imaging Laboratory, London, United Kingdom) using proportional scaling, setting the height threshold at *P*≤0.001 and cluster size threshold at >100 voxels, and including group (C-LHPA *vs*. LPA) as a covariate.

### Representativeness

#### Physical aggression trajectory classification

The average posterior probability for the assigned trajectory group for the current study was 0.73 and 0.85 for the two trajectories characterized by low levels of aggression (LPA group, 69% of the sample) and 0.87 for the trajectory characterized by high levels of aggression in childhood, that started to decline at the end of elementary school (C-LHPA, 28% of the sample). These values are in agreement with the mean posterior probability for the assigned groups, when calculated over the entire cohort (*N* = 1,037) (0.73, 0.90, 0.83, respectively) [3]. This statistic ensures representativeness of the sample studied for each trajectory.

#### Family adversity

We compared the two groups who participated in the present study (C-LHPA, LPA) to the participants who did not. Within the high C-LHPA group individuals who took part in the study demonstrated a less adverse childhood environment (mean ± s.e.m.: 0.23±0.05 *vs*. 0.44±0.01; (F(1,318) = 6.74, *P* = 0.01) than those who did not. A closer inspection of the data showed that this was particularly true for early childhood adversity (age 6–10) (mean ± s.e.m.: 0.23±0.05 vs. 0.44±0.01 (F(1, 316) = 5.84, *P* = 0.016), and late childhood adversity/early adolescence (age 11–14) (mean ± s.e.m.: 0.23±0.04 *vs*. 0.44±0.01; F(1,257) = 5.12, *P* = 0.024), but not for adversity in late adolescence (age 15–16) (mean ± s.e.m.: 0.30±0.08 *vs*. 0.45±0.01). Within the LPA group there were no differences in childhood adversity levels between the participants and non-participants.

#### Current psychosocial functioning

For both the C-LHPA and LPA groups, there were no group differences in psychosocial functioning, including civil status, number of children, health, education level, job status, average salary, number and quality of relationships, criminal status, and quality of intimate relationships between those who took part *vs*. those who did not.

## Supporting Information

Table S1 Comparison of psychosocial and demographic variables between the LPA and C-LHPA developmental trajectory groups.
(0.06 MB DOC)
