# Supplementary material for: Brain Serotonin Synthesis in Adult Males Characterized by Physical Aggression during Childhood: A 21-Year Longitudinal Study
Source: PLoS One. 2010 Jun 22;5(6):e11255. doi: 10.1371/journal.pone.0011255 (PMC2889822; doi:10.1371/journal.pone.0011255)
Supplement: Table S1 — Comparison of psychosocial and demographic variables between the LPA and C-LHPA developmental trajectory groups. (0.06 MB DOC) [file pone.0011255.s001.doc]

**Table S1**. **Comparison of psychosocial and demographic variables between the LPA and C-LHPA developmental trajectory groups.**

| *Variable* | LPA  (*n*=18) | C-LHPA  (*n*=8) | *P*-value |
| --- | --- | --- | --- |
| **Sociodemographic factors:**  *Civil Status:* Bachelor / Married or Common law / Divorced (%)  *Having Children* (yes / no) (%) | 60 / 33 /7  27 / 63 | 50 / 50 / 0  17 / 83 | 0.67  0.55 |
| **Health :**  Self reported health evaluation (range : 1-5; 1=excellent-5=bad) | 2.1 (0.2) | 2.7 (0.2) | 0.104 |
| Use of health and law services the past year  (8 items, possible range 0-24; 0=never, 3=regularly) | 2.1 (0.5) | 2.3 (0.4) | 0.87 |
| Ever hospitalized for at least 1 week (detention, psychiatric hospital, physical rehabilitation, intoxication,..) (yes / no) | 20% / 80% | 17% / 83% | 0.68 |
| Smoker (%) | 22.2% | 25% | 0.62 |
| **Education:** |  |  |  |
| Years of education (s.e.m.) ^1^ | 6.7 (0.6) | 6.9 (1.3) | 0.97 |
| Highest completed Education level (%): |  |  |  |
| Kindergarten / Elementary School | 11% | 12.5% | 0.99 ^2^ |
| Highschool | 39% | 37.5% |  |
| College / Pre-university | 39% | 25% |  |
| University Degree (BSc / BA) (aggregate as in analysis) | 11% | 25% |  |
| **Work:** |  |  |  |
| Has a fulltime job the past 12 months (%)  Job ranking (past y)  Salary / hour in $ (past y) | 87%  39.3 (2.3)  17.6 (1.8) | 100%  39.9 (5.3)  18.1 (1.9) | 0.50  0.91  0.95 |
| Received Welfare or CSST between age 20-28 (yes / no) (%) | 20 / 80 | 0 / 100 | 0.34 |
| Received Social help or CSST at age 28 (current) (yes / no) (%) | 6.7 / 93.3 | 0 / 100 | 0.71 |
| **Social functioning :** |  |  |  |
| Social activities with family or friends (range : 4-20; 4=every week; 20=never) | 7.4 (0.6) | 7.3 (1.1) | 0.91 |
| Involvement in organizations such as recreational, sport, cultural, political (possible range 7-14; 7=yes; 14=no) | 13.4 (0.2) | 13.2 (0.5) | 0.56 |
| How many friends ever arrested by police? | 1.1 (0.1) | 1.2 (0.2) | 0.88 |
| Has closest friend ever been arrested by police? | 13.3% | 16.7% | 0.66 |
| Self-reported delinquent acts the past 12 months  (33 items, 1=never, 4=often; range : 33-132) | 35.1 (1.3) | 36.8 (3.0) | 0.53 |
| How many adults do you know who have done crimes?  None  1 or 2  Some  Quite a few | 20%  40%  33.3%  6.7% (n=1) | 16.7 %  16.7%  50.0%  16.7% (n=1) | 0.68 |
| Got a ticket for driving the past year :  Never  1-2 times  A few times  Is not allowed to drive any more | 60%  33%  7%  0% | 50%  33%  0%  17% (n=1) | 0.39 |
| **Love relationships :** |  |  |  |
| -Is currently in a relationship (yes / no) (%)  -How many months currently in relationship (s.e.m.)  -Current partner has been arrested by the police the past 12 months (yes / no) (%)  -Tension / fighting in current relationship (13 items, range 0-39; 0= never, 3=very often) | 60 / 40  35.2 (10.8)  0 / 100  1.4 (0.4) | 67 / 33  34.7 (13.7)  0 / 100  2.5 (1.3) | 0.59  0.99  1.00  0.35 |

*Note*. Values represent the raw (uncorrected) means (s.e.m.) or percentages, whatever applicable. *P* values are corrected for probability of trajectory, whenever applicable. Values (except for smoking and education; *N*=26) are missing for 5 participants (2 C-LHPA, 3 LPA). *P*-values represent chi-square or one tailed F-tests, whatever applicable.

^1^ measured from the start of highschool ^2^ Chi-square calculations are based on a 3X2 table (high vs. low physical aggression X elementary school or less, high school, higher than highschool).

LPA= Normal development of low physical aggression

C-LHPA= Childhood-limited high physical aggression
